# The Chinese Herb Yi-Qi-Huo-Xue Protects Cardiomyocyte Function in Diabetic Cardiomyopathy

**DOI:** 10.1155/2018/7316840

**Published:** 2018-05-06

**Authors:** Xiangsheng Wang, Jing Huang, Shengyi Wang, Qing Ni

**Affiliations:** ^1^Jining Traditional Chinese Medicine Hospital, Jining, Shandong 273100, China; ^2^Beijing No. 1 Hospital of Integrated Traditional Chinese and Western Medicine, Beijing 100026, China; ^3^Xinhua College of Sun Yat-sen University, Guangzhou, Guangdong 510520, China; ^4^Guang'anmen Hospital, China Academy of Chinese Medical Sciences, Beijing 100053, China

## Abstract

*Aims. *To study the effect of the Chinese herb Yi-qi-huo-xue on cardiomyopathy in diabetic rats.* Methods*. Rats were fed a high fat and high glucose diet and injected with 50 ml/kg streptozotocin (STZ) to induce diabetic cardiomyopathy (DCM), followed by treatment with Yi-qi-huo-xue for 4 weeks. We measured the rats' heart weight index, observed the myocardial morphology using hematoxylin eosin (HE) staining, and determined the content of collagen types I and III in the myocardium using enzyme-linked immunosorbent assay (ELISA). We determined Bcl-2, Bax, and P53 protein expression by Western blot analysis and the cardiomyocyte apoptosis rate via a flow cytometry assay.* Results.* Compared with the rats in the control group, the diabetic rats gained weight and had increased blood sugar levels, an enhanced heart weight index, and increased myocardial pathophysiological damage. There was a decrease in their Bcl-2 expression, and their Bax and P53 expression increased. The Bcl-2/Bax ratio was enhanced, and there was an increase in the content of collagen types I and III in the myocardium. After treatment with Yi-qi-huo-xue, all levels listed above returned to normal.* Conclusion. *The Chinese herb Yi-qi-huo-xue degraded the myocardial interstitial collagen types I and III to protect the myocardium of the diabetic rats, thus delaying the role of myocardial fibrosis. Yi-qi-huo-xue could play an important role in protecting the myocardium of DCM rats by enhancing the expression of the Bcl-2 protein, inhibiting the expression of the Bax and P53 proteins, increasing the ratio of Bcl-2/Bax, and inhibiting the apoptosis of cardiomyocytes.

## 1. Introduction

Diabetes is a global health concern [1], and in 2014, 422 million people had the disease. It is the leading cause for the pandemic rise in heart disease complications [2, 3]. They develop cardiovascular diseases including heart failure and diabetic cardiomyopathy (DCM) at rates three to five times higher than that of the general population [4, 5]. Diabetic cardiomyopathy is one of the major causes for end-stage heart failure. There are 3 stages in its pathophysiology: the early stage includes cardiac hypertrophy and diastolic dysfunction, the second stage occurs with the thickening of the left ventricular wall, myocardial cell apoptosis, and myocardial fibrosis, and the third stage involves contraction dysfunction, arrhythmia, heart failure, and even sudden death [6–10]. The molecular and cellular mechanisms responsible for diabetic cardiomyopathy are complicated and result in multiple issues, including metabolic abnormalities, myocardial fibrosis, microvascular disease, insulin resistance, oxidative stress, and apoptosis [11]. The morphology of DCM includes cardiomyocyte hypertrophy, necrosis, extracellular matrix deposition, myocardial fibrosis, and cardiomyocyte apoptosis [12]. In clinical treatment, there are no specific pharmacological agents or therapeutic strategies that slow the progression of myocardial fibrosis and cardiomyocyte apoptosis [13].

In China, a long history of clinical practice shows good clinical results when Yi-qi-huo-xue is used to treat diabetes and its accompanying cardiovascular complications, even cardiovascular disease [14]. In this study, Yi-qi-huo-xue herb is from Shengmai San and Danshen Decoction which are two classical complex prescriptions of traditional Chinese medicine (TCM). It is widely used for cardiovascular complications in diabetes mellitus and cardiovascular disease and found to be both of the highest frequency of use and of thebest effect by data-mining on 1274 clinical cases of multicentered, diabetic in-patients with coronary heart disease [15]. Labs also found that the herb possessed extensive pharmacological activities, such as improvement of the LVEF (left ventricular ejection fraction) and the TCM (Traditional Chinese Medicine) syndrome scores [16]; However, to date, the mechanisms responsible for its effectiveness in individuals with DCM are poorly understood. Thus, the purpose of this study was to assess whether the effects of myocardial disease in DCM rats were reversed by Yi-qi-huo-xue.

## 2. Materials and Methods

### 2.1. Overview of the Experiments

**Figure 1:**
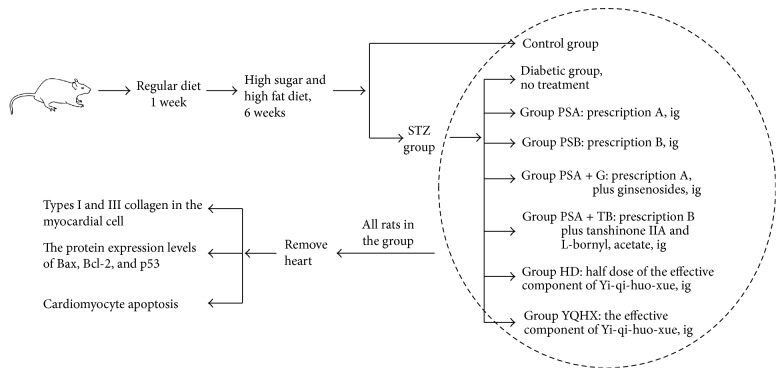
Overview of the experiments.

We assessed the mechanism of Yi-qi-huo-xue in treating DCM in a diabetic rat model. The Sprague Dawley (SD) rats were subjected to streptozotocin- (STZ-) induced diabetes; following the Yi-qi-huo-xue herbal treatment, the rats were placed under deep anesthesia, and their hearts were rapidly removed, stored in −80°C until use. We tested the levels of collagen types I and III in the myocardial cells, the protein expression levels of Bax, Bcl-2, and P53, and the cardiomyocyte apoptosis rate. A schematic of the study design is shown in Figure 1.

### 2.2. Reagents

Yi-qi-huo-xue sample (Renshen 9 g, Maodong 9 g, Wuweizi 6 g, Danshen 30 g, Tanxiang 6 g, and Sharen 6 g) was obtained from Guang'anmen Hospital at the China Academy of Chinese Medical Sciences. The sample was decocted in water to a concentration of 0.95 g/ml and stored at 4°C. The effective components of Yi-qi-huo-xue (ginsenosides, Ophiopogon japonicus polysaccharide, Schisandra rosea oil, tanshinone IIA, sandalwood oil, and L-bornyl acetate) were obtained from Nanjing Zelang Pharmaceutical Technology Co., Ltd. (Nanjing, Jiangsu, China); the STZ and SDS were purchased from Sigma-Aldrich (St. Louis, MO). Bax, Bcl-2, anti-P53 antibody, the Animal-Plant Total Protein Miniprep Kit, Chromogenic AP, and BCA Protein Assay Kit were purchased from Beijing Tianze Genetic Science and Technology Ltd., Co. (Beijing, China). All other solvents and reagents used were of the highest grade commercially available.

### 2.3. Induction and Verification of Type 1 Diabetes

SD rats (200–220 g) were used in this investigation and were approved by the Institutional Animal Care and Use Committee of Guang'anmen Hospital at the China Academy of Chinese Medical Sciences. SD male rats were purchased from Beijing Vital River Laboratory Animal Technology Co., Ltd. (Beijing, China). The rats were given free access to water and food and housed at room temperature (20°C–25°C) with a relative humidity of 40%–60% under natural light.

### 2.4. Treatment of Diabetic Animals

All experiments were approved by the Local Ethics Committee for Animal Research Studies at the Guang'anmen Hospital. Fifty SD rats were given a regular diet including protein (23%), carbohydrates (53%), and fat (5%). After 1 week, the rats were given a high sugar and high fat diet, which included a regular diet (66.5%), lard (10%), cholesterol (53%), bile salts (1%), and sucrose (20%) for 4 weeks. The rats then fasted for 12 hours before their tail vein blood was collected and blood sugar level was tested. After this, the rats were given a high sugar and high fat diet; then 6 rats were randomly chosen and injected with a citrate buffer to serve as controls, another 44 rats were injected with freshly prepared STZ (50 mg/kg) with a cold 0.1 M citrate buffer, and we tested their blood sugar levels at 1 day, 3 days, and 7 days. Rats that showed a blood glucose level of >16.7 mmol/L twice and that had clinical symptoms such as polyphagia, polydipsia, polyuria, and weight loss following the STZ injection were considered to be diabetic [17]. The diabetic rats were randomly divided into 5 group: group PSA: prescription A, 10 ml/kg·d, ig.; group PSB: prescription B, 10 ml/kg·d, ig.; group PSA + G: prescription A plus ginsenosides, 10 ml/kg·d, ig.; group PSB + TB: prescription B plus tanshinone IIA, L-bornyl acetate, 10 ml/kg·d, ig.; group HD: half dose of the effective component of Yi-qi-huo-xue, 10 ml/kg·d, ig.; and group YQHX: Yi-qi-huo-xue 7.5 ml/kg·d, ig. The details administered doses for the groups are shown in Table 1. The doses were administered for 4 weeks on a dosing schedule of once a day, 6 times a week.

### 2.5. Collection of Tissue Samples

The rats were given the treatment for 4 weeks and then fasted for 12 hours before their blood sugar levels were tested. After euthanasia using 1% sodium pentobarbital (2 ml/kg, ip.), their chest cavities were opened, and the hearts were rapidly removed and placed in a cold isotonic NaCl buffer, dried with filter paper, and weighed. The left ventricle was cut into 3 parts: one part was immersed in paraformaldehyde for 24 hours at 4°C and the other parts were placed in liquid nitrogen until use. The rat heart weight index was the heart weight (mg)/body weight.

### 2.6. HE Staining

Transverse 8.0 *μ*m thick serial sections were cut from paraffin-embedded LV slices and mounted. Hematoxylin and eosin (HE) staining was used for the general morphological evaluation [18].

### 2.7. Enzyme-Linked Immunosorbent Assay (ELISA)

The levels of types I and III collagen in the myocardial cell lysis buffer and supernatants were measured using the ELISA assay kits. The OD values were measured at 450 nm with an ELX800 microplate reader.

### 2.8. Flow Cytometry Proliferation Assay

The left ventricle was cut and homogenized in a cold PBS buffer and centrifuged at 1000 rpm for 5 minutes. The suspension with 400 ul 1x binding buffer was centrifuged at 1000 rpm for 5 minutes. The count and suspension cell were 1 × 10^6^/ml. We then added 5 uL Annexin V-FITC and incubated the samples at 4°C for 30 minutes before adding 10 *μ*l PI. We then incubated the samples at 4°C for 30 minutes and tested the cardiomyocyte apoptosis rate using flow cytometry.

### 2.9. Western Blot Test

Western blotting assays were used to measure the protein expression levels of Bcl-2, Bax, P53, and *β*-actin in the left ventricle. 100 mg of the left ventricular tissue was lysed in a 1 ml lysis buffer and homogenized on ice. Protein concentrations were assessed using a bicinchoninic acid protein assay kit (Dingguo, Beijing, China). Equal amounts of protein were subjected to 12% SDS polyacrylamide gel electrophoresis and transferred onto PVDF membranes using a Bio-Rad Western blot analysis apparatus. Then, the membranes were blocked in 5% nonfat dry milk in PBST and incubated with P53, Bax, Bcl-2 (1 : 1000 dilution), and *β*-actin (1 : 2000) antibodies (Cell Signaling Technology, Danvers, MA) overnight at 4°C. After being washed 3 times with PBST, the membranes were incubated with the corresponding secondary antibodies (1 : 6000 dilution, Sigma, MS, USA) for 2 hours at room temperature. The immunolabeled bands were visualized using a Pierce ECL Western blotting substrate (Millipore, Bedford, USA).

### 2.10. Statistical Analysis

All data are presented as the mean ± SEM. The data were evaluated using one-way ANOVA, and the between-group comparisons were performed using LSD *t*-test employing SPSS 20.0 (IBM Corp., Armonk, NY).

## 3. Results

### 3.1. Establishment of General Clinical Symptoms in the Diabetic Model

The control group had normal drinking and eating habits, polyuria, weight, walks, and responses, their lips and claws were ruddy, and they had good hair with bright coloration; the rats had also no lesions or bleeding. The diabetic rats had polyphagia, polydipsia, polyuria, and weight loss, they exhibited decreased activity and were unresponsive, their lips and claws were pale, their hair color was dull, and their hair came off easily, and these rats had skin lesions and tail bleeding. The clinical symptoms improved in the treatment group, after treatment for 4 weeks, the blood sugar level in the control group was 5.78  ±  0.57 and 31.07  ±  1.27 in the diabetic group. Compared with the control group, this was a 5.38-fold increase (*P* < 0.01). Compared with the diabetic group, all 6 groups showed an increase from 26.43 ± 1.80 (group PSA) to 29.45 ± 1.29 (group PSA + G), and groups PSA, PSB, and YQHX had lower values than groups PSA + G, PSB + TB, and HD (*P* < 0.05, Table 2).

### 3.2. Heart Weight/Body Weight Ratios

The heart weight/body weight ratio in the control group was 2.43 ± 0.24 and 3.09 ± 0.05 in the diabetic group, and all 6 groups decrease the ratio range from 2.73 ± 0.21 to 2.96 ± 0.38 (Table 3).

### 3.3. HE Staining

**Figure 2:**
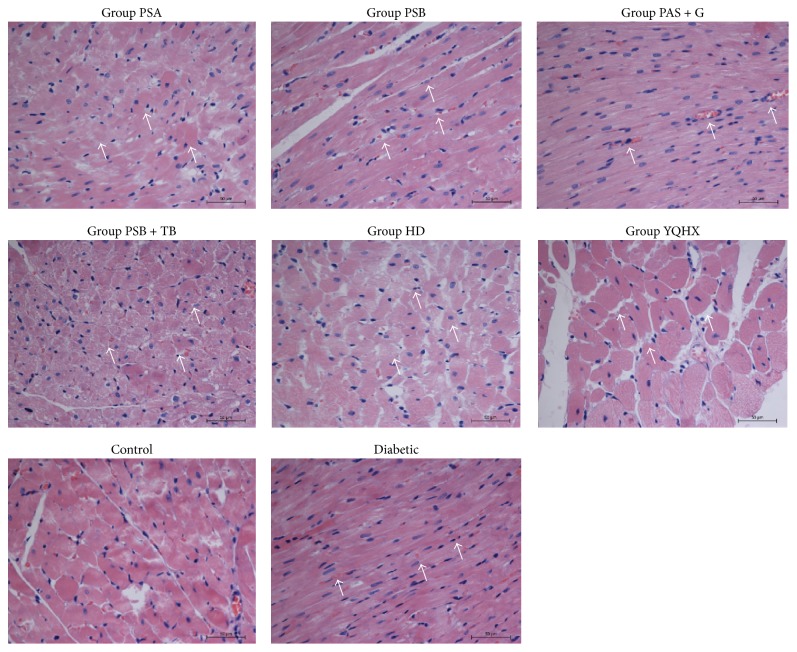
The normal, diabetic, and treatment groups' myocardia were stained with hematoxylin eosin. The diabetic cardiac muscle fibers were disordered and many of them had collapsed. The diabetic myocardium showed fibrosis and extensive focal coalescent areas of ischemic myocyte degeneration in the subendocardial and subepicardial regions and inflammatory cell infiltration (Bar = 50 *μ*m).

The rats' cardiac tissue and cardiomyocytes had a normal size and morphology, with muscle fibers arranged in neat rows; the cytoplasm and nuclear staining was uniform with no cell body edema or inflammatory cell infiltration. The diabetic cardiac tissue and cardiomyocytes showed an irregular shape, cell hypertrophy, a cell gap increase, myocardial fiber disarrangement, muscle fiber fracture, myocardial cell edema, and inflammatory cell infiltration. The treatment group showed fewer myocardial cells with edema and necrosis and less muscle fiber disarrangement compared with the diabetic group (see the white arrows in Figure 2).

### 3.4. Enzyme-Linked Immunosorbent Assay (ELISA)

The ELISA values for normal myocardial collagen types I and III were 4.27 ± 0.02 and 6.66 ± 0.08, respectively, and diabetic myocardial collagen types I and III were 5.61 ± 0.49 and 11.90 ± 1.21, respectively. Group PSB had decreased myocardial collagen types I and III compared with the diabetic group (*P* < 0.05, *P* < 0.01). The myocardial collagen I values of groups PSA and PSB were 4.36 ± 0.47 and 4.55 ± 0.47, respectively, a lower level of myocardial collagen I compared with the diabetic group (*P* < 0.05). The myocardial collagen III in groups PSA and YQHX had values of 7.91 ± 0.11 and 8.67 ± 0.06, respectively, and had lower myocardial collagen III levels compared with the diabetic group (*P* < 0.01, *P* < 0.05). The myocardial collagen III in Groups PSA + G and PSB + TB had values of 10.12 ± 0.41 and 9.86 ± 0.77, respectively; these groups had more myocardial collagen III than groups PSA (*P* < 0.01) and PSB (*P* < 0.05, Table 4).

### 3.5. Flow Cytometry Proliferation Assay

**Figure 3:**
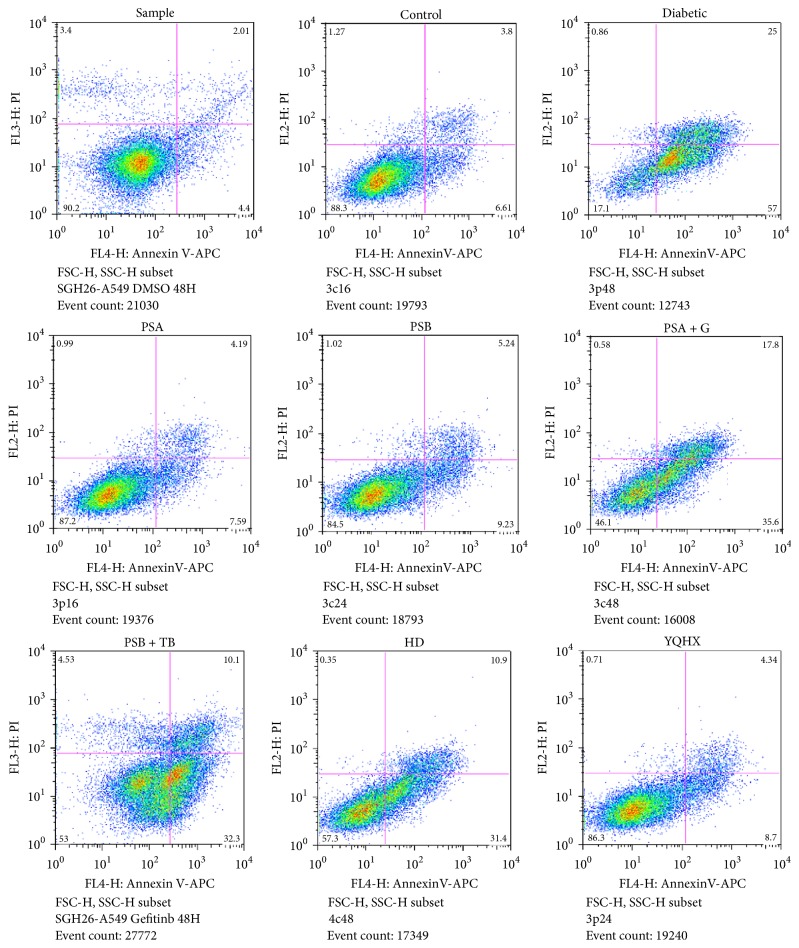
The flow cytometry assay shows 4 areas. Q1: (AnnexinV− FITC)^−^/PI^+^, necrotic cells or a few late apoptotic cells, mechanically damaged cells; Q2: (AnnexinV+ FITC)^+^/PI^+^, late apoptotic cells; Q3: (AnnexinV− FITC)^+^/PI^−^, early late apoptotic cells; Q4: (AnnexinV− FITC)^−^/PI^−^, live cell. Q3 + Q2 = apoptotic cells ratio. Compared with the control group, groups PSA + G, PSA + TB, and HD had enhanced diabetic myocardial cell apoptotic rates (*P* < 0.01). Compared with the diabetic group, the myocardial cells apoptotic rate decreased in groups PSA, PSA, PSA + G, HD, and YQHX (*P* < 0.01). Compared with group PSA, there was a decrease the myocardial cell apoptotic rate in groups PSA + G, PSA + TB, and HD (*P* < 0.05). Compared with group PSB, there was a decrease in the myocardial cell apoptotic rate in group PSB + TB (*P* < 0.05).

The flow cytometry proliferation assay showed that the normal myocardial cells' Q2 + Q3 apoptotic rate was 6.27 ± 0.15% diabetic group (53.53 ± 31.20, *P* < 0.01), group PSA + G (31.66 ± 11.77%, *P* < 0.01), group PSB + TB (37.23 ± 17.72%, *P* < 0.01), group HD (30.97 ± 12.42%, *P* < 0.01), group PSA (13.78  ±  2.19%, *P* < 0.05), group PSB (16.32 ± 2.03%, *P* < 0.05), and group YQHX (15.26  ±  2.43%, *P* < 0.05). Compared with the diabetic group, groups PSA, PSB, PSB + TB, HD, and YQHX experienced decreases in their apoptotic rates (*P* < 0.05). There were no significant differences between groups PSA, PSB, and 6 (*P* > 0.05). Group PSA's apoptotic rate was significantly decreased compared with groups PSA + B, PSB + TB, and HD (*P* < 0.05). Compared with group PSB + TB, the apoptotic rate in group PSB was decreased (*P* < 0.05) (Figure 3, Table 5).

### 3.6. Western Blot Test

**Figure 4:**
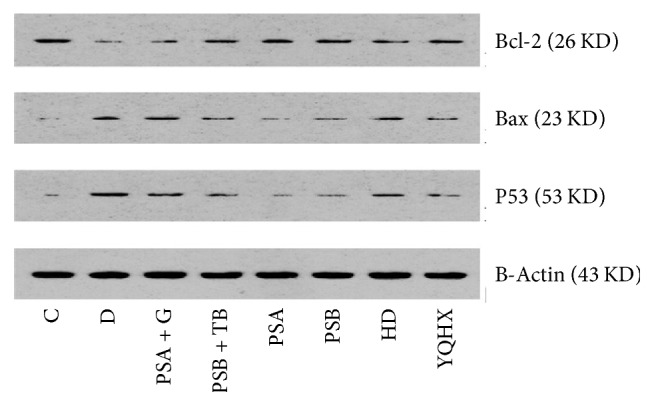
Compared with the control group, the diabetic myocardial Bcl-2 levels decreased, the Bax and P53 levels increased, and Bcl-2/Bax decreased. Compared with the diabetic group, all treatment groups' Bcl-2 levels increased, their Bax and P53 levels decreased, and Bcl-2/Bax increased.

Compared with the control group, the diabetic group's myocardial Bcl-2 levels decreased, its Bax and P53 levels increased, and its Bcl-2/Bax levels decreased. Compared with the diabetic group, all treatment groups' Bcl-2 levels increased, the Bax and P53 levels decreased, and the Bcl-2/Bax levels increased (Figure 4).

## 4. Discussion

Chinese herbal medicines are part of major therapeutic strategies and have thousands of years of history. While the treatment effects are clear, the underlying mechanisms are under considerable debate. It is becoming clearer that Yi-qi-huo-xue contributes to the treatment of DCM and improves the clinical symptoms of the disease, including polyphagia, polydipsia, polyuria, weight loss, and blood sugar levels after 4 weeks of treatment; for example, our data show that the diabetics had decreased body weight by 0.59-fold, increased blood sugar level by 5.38-fold, and heart weight/body weight ratios of 2.27; the data show the significant change in diabetics that have important improvement in signs and clinical symptoms after treatment with Yi-qi-huo-xue herb. This indicates that Yi-qi-huo-xue herb protects the cardiovascular muscle via bringing down blood sugar level and losing body weight. However, what are the mechanisms?

The principal finding of the present study is that Yi-qi-huo-xue decreased the myocardial cells' apoptotic rate. Myocardial cell apoptosis is an important pathophysiological mechanism in DCM. Apoptosis, also known as programmed cell death, plays an important role in maintaining the normal morphology and function of tissues and organs; DCM researcher has focused on the protein expression of apoptosis-related genes in cardiomyocytes [19–21]. The Bcl-2 family of proteins plays an important role in the process of apoptosis. Their role in apoptosis is divided into actions of antiapoptotic proteins and proapoptotic proteins. The antiapoptotic proteins include Bcl-2, Bcl-xL, Bcl-w, Mcl-1, A1, and Boo. The proapoptotic proteins include Bax, Bak, Bok, Bcl-xS, Bid, and Bad. The balance of these two kinds of proteins working together determines whether cells enter the apoptotic process. Bcl-2 and Bax are the most representative of the Bcl-2 family in that they inhibit apoptosis and promote apoptosis, respectively. Bax is the main regulator of Bcl-2 activity, and Bcl-2 inhibits apoptosis by interacting with the mitochondria. Bax induces apoptosis by modulating the mitochondria [22]. The tumor suppressor gene P53 can activate its downstream pathway to regulate a large number of cellular activities that play important roles in preventing cell proliferation and maintaining the integrity of DNA-damaged genomes [23]. In this study, we observed the expression of an apoptosis-related factor protein in the myocardia of rats with DCM. In the diabetic rats, Bcl-2 decreased, Bax and P53 expression increased, and the Bcl-2/Bax ratio decreased. After treatment with Yi-qi-huo-xue, the myocardial cell Bcl-2 increased, Bax and P53 expression decreased, and the Bcl-2/Bax ratio increased. These are very important results, as they suggest that Yi-qi-huo-xue is involved in preventing anticardiomyocyte apoptosis via increased Bcl-2, inhibiting Bax and P53 levels, and an increased Bcl-2/Bax ratio.

Parallel studies also found that Yi-qi-huo-xue could play a role in protecting against myocardial fibrosis in diabetic rats via the degradation of myocardial interstitial collagen types I and III. Our data showed that diabetic myocardial collagen types I and III increase by 1.31-fold and 1.79-fold compared to control group, respectively; after treatment of Yi-qi-huo-xue, the myocardial collagen types I and III is 0.41, 1.3-fold compared to control group; the HE stain data showed diabetic cardiomyocyte hypertrophy, myocardial fiber rupture, dissolution, necrosis, and inflammatory cell infiltration. This is consistent with previous reports of diabetic myocardial pathology [24–26]. In fact, cardiomyocyte apoptosis and myocardial fibrosis promote each other. Myocardial fibrosis leads to reduced ventricular wall elasticity and increased diastolic pressure and pressure on the myocardial cell load stimulation, resulting in vascular fibrosis that affects the coronary microcirculation, interstitial fibers (leading to tissue oxygen diffusion disorder), and hypoxia (an important predisposing factor in myocardial cell apoptosis). In return, cardiomyocyte apoptosis is not only an important mechanism of cardiac dysfunction and decreased cardiac function, it also increases the intraventricular pressure load. Researchers have confirmed that myocardial cell apoptosis itself is the cause of myocardial fibrosis [27–29]. Our data suggest that Yi-qi-huo-xue improved cardiac function and delayed myocardial fibrosis in diabetic rats via the degradation of myocardial interstitial collagen.

In conclusion, we have shown that using the Chinese herb Yi-qi-huo-xue improved the blood sugar level in rats and that Yi-qi-huo-xue could degrade myocardial interstitial collagen types I and III to protect the myocardia of diabetic rats, delaying the role of myocardial fibrosis. We also showed that an extract of Yi-qi-huo-xue had the same effects as the herb in controlling the blood sugar level and reducing myocardial cell apoptosis. However, the effect is reduced by adding or deleting the active component. Chinese herbs could play an important role in protecting the myocardia of DCM rats by enhancing the expression of the Bcl-2 protein, inhibiting the expression of Bax and P53, increasing the ratio of Bcl-2 and Bax, and inhibiting the apoptosis of cardiomyocytes. These new data indicate that Yi-qi-huo-xue can improve the mechanism of cardiomyopathy in diabetic rats and provide a therapeutic method for clinical treatment.

## Figures and Tables

**Table 1:** Overview of groups and treatment.

Group	Dose (g/kg·d)					
	Ginsenosides	Ophiopogon japonicus polysaccharide	Schisandra rosea oil	Tanshinone IIA	Sandalwood oil	L-bornyl acetate
PSA	0	1.35	0.585	0.27	0.54	0.54
PSB	2.7	1.35	0.9	0	0.315	0
PSA + G	1.485	1.35	0.585	0.27	0.54	0.54
PSB + TB	2.7	1.35	0.9	1.485	0.315	0.315
HD	1.485	0.945	0.585	1.485	0.315	0.315
YQHX	Yi-qi-huo-xue, 7.5 ml/kg·d					
Control	Citrate buffer					
Diabetic	Citrate buffer					

*Note*. Groups PSA and PSB have better prescriptions than the other groups.

**Table 2:** Rats' weights and blood sugar levels (mean ± SD).

Group	*n*	Weight (g)	Blood sugar level (mmol/l)
PSA	7	448.67 ± 36.55^*∗∗*##^	26.43 ± 1.80^*∗∗*##^
PSB	6	459.50 ± 31.07^*∗∗*##^	26.73 ± 0.91^*∗∗*##^
PSA + G	6	373.67 ± 20.02^*∗∗*#^	29.45 ± 1.29^*∗∗*#^
PSB + TB	6	370.50 ± 19.04^*∗∗*#^	29.27 ± 1.18^*∗∗*#^
HD	6	390.17 ± 27.24^*∗∗*##^	28.65 ± 1.06^*∗∗*##^
YQHX	6	439.33 ± 25.83^*∗∗*##^	27.28 ± 0.77^*∗∗*##^
Control	6	557.67 ± 21.12^##^	5.78 ± 0.57^##^
Diabetic	5	329.33 ± 33.12^*∗∗*^	31.07 ± 1.27^*∗∗*^

*Note*. Compared with the control group; ^*∗∗*^*P* < 0.01. Compared with the diabetic group; ^#^*P* < 0.05; ^##^*P* < 0.01.

**Table 3:** Heart weight/body weight ratios (mean ± SD).

Group	Heart weight/body weight ratios
Control group	2.43 ± 0.24
Diabetic group	3.09 ± 0.05^*∗∗*^
PSA	2.73 ± 0.21
PSB	2.86 ± 0.45
PSA + G	2.96 ± 0.38
PSB + TB	2.79 ± 0.61
HD	2.84 ± 0.31
YQHX	2.76 ± 0.29

*Note*. Compared with the control group; ^*∗∗*^*P* < 0.01.

**Table 4:** Concentration of myocardial collagen types I and III (mean ± SD).

Group	Collagen I (ng/ml)	Collagen III (ng/ml)
PSA	4.36 ± 0.47^#^	7.91 ± 0.11^*∗*##^
PSB	4.55 ± 0.47^#^	8.46 ± 0.01^*∗∗*##^
PSA + G	5,16 ± 0.48	10.12 ± 0.41^*∗∗*#^
PSB + TB	5.01 ± 0.48	9.86 ± 0.77^*∗∗*##^
HD	4.89 ± 0.47	9.07 ± 0.02^*∗∗*##^
YQHX	4.77 ± 0.48	8.67 ± 0.06^*∗∗*##^
Control group	4.27 ± 0.02^#^	6.66 ± 0.08^##^
Diabetic group	5.61 ± 0.49^*∗*^	11.90 ± 1.21^*∗∗*^

*Note*. Compared with the control group, ^*∗*^*P* < 0.05, ^*∗∗*^*P* < 0.01. Compared with the diabetic group, ^#^*P* < 0.05, ^##^*P* < 0.01. Compared with group A.

**Table 5:** Myocardial cells' apoptotic rates (mean ± SD).

Group	*n*	Q2 + Q3 apoptotic rate (%)
PSA	7	13.78 ± 2.19^##^
PSB	6	16.32 ± 2.03^##^
PSA + G	6	31.66 ± 11.77^*∗∗*#^
PSB + TB	6	37.23 ± 17.72^*∗∗*^
HD	6	30.97 ± 12.42^*∗∗*##^
YQHX	6	15.26 ± 2.43^##^
Control group	6	6.27 ± 0.15^##^
Diabetic group	5	53.53 ± 31.20^*∗∗*^

*Note*. Compared with the control group; ^*∗∗*^*P* < 0.01. Compared with the diabetic group; ^#^*P* < 0.05; ^##^*P* < 0.01.
